# Beyond the Snare: Utilizing Rigid Laparoscopic Forceps for the Retrieval of a Wall-Embedded Inferior Vena Cava (IVC) Filter

**DOI:** 10.7759/cureus.106514

**Published:** 2026-04-06

**Authors:** Saleem Jahangir, Mohammed S Algahtani, Khalid Alomar, Abdullah Alfozan, Bader Alsuwailem, Majdi Ibrahim, Saad Bin Ayeed, Tariq Wani, Abdulwali Alwan

**Affiliations:** 1 Vascular Surgery, King Fahad Medical City, Riyadh, SAU; 2 General Surgery, Imam Muhammed Ibn Saud Islamic University, Riyadh, SAU

**Keywords:** advanced endovascular technique, case report, filter embolization, ivc filter retrieval, rigid laparoscopic forceps, suprarenal ivc filter

## Abstract

Retrievable inferior vena cava (IVC) filters are commonly placed to prevent pulmonary embolism when anticoagulation is contraindicated. Although guidelines strongly recommend timely removal, many filters become firmly embedded within the caval wall, rendering standard snare retrieval impossible. Advanced techniques are then needed to avoid open surgery.

A 43-year-old male with a history of asthma, sleeve gastrectomy, and recent cholecystectomy had a retrievable IVC filter placed four months prior due to a minor IVC injury sustained during surgery. He presented for elective removal. Under general anesthesia in a hybrid operating room, initial attempts with a snare, ureteroscopic forceps, and Fogarty balloon dissection all failed owing to the firm embedding of the filter hook and struts. Rigid laparoscopic forceps were then introduced through a large-bore sheath. The strong jaws securely captured the filter tip and allowed careful detachment of each embedded strut. The filter was removed in one piece. Completion angiography revealed no extravasation. The patient recovered well and was discharged the next day without requiring pain medication.

Rigid laparoscopic forceps provide a safe, effective, and readily available bail-out tool for complex embedded IVC filter retrieval when conventional and flexible endovascular techniques fail, even with short dwell times. This approach achieves high success rates with minimal morbidity and avoids the need for open surgical conversion.

## Introduction

Inferior vena cava (IVC) filters are endovascular devices placed to prevent pulmonary embolism in patients with acute venous thromboembolism when anticoagulation is contraindicated or has failed. These devices are inserted percutaneously into the IVC to capture emboli originating from the lower extremities and pelvis [1]. Since the introduction of retrievable IVC filters in the early 2000s, clinicians have preferred them over permanent filters because they can be removed once the acute risk period has passed and anticoagulation is safe [1, 2]. Professional societies recommend retrieval when the device is no longer indicated [3].

Long-term indwelling filters carry rising risks over time. Complications include caval penetration, filter fracture, component migration, IVC thrombosis, and higher rates of lower extremity deep vein thrombosis. Penetration can cause chronic pain or injury to adjacent structures such as the duodenum or aorta. Thrombosis may result in post-thrombotic syndrome [1, 4]. In 2010, the U.S. Food and Drug Administration (FDA) issued a safety alert regarding adverse events from prolonged implantation of retrievable filters. The agency updated this guidance in 2014, recommending removal once the risk of pulmonary embolism has resolved. Some decision analyses have suggested that the optimal retrieval window may be between 29 and 54 days, but this should be interpreted as a general guideline, and the timing of retrieval should ultimately be individualized based on each patient's clinical circumstances and risk-benefit assessment [3].

Despite these recommendations, retrieval rates remain disappointingly low worldwide, with reported rates as low as 8.5% and a mean of 34% in a large institutional series [3]. Barriers include loss to follow-up, lack of systematic tracking, patient comorbidities, and perceived technical difficulty by referring physicians. Consequently, many filters become permanent by default, exposing patients to unnecessary long-term complications [4]. Prolonged dwell time promotes endothelialization and fibrotic incorporation of the filter hook and struts into the caval wall, which significantly complicates removal [3, 4].

Common advanced retrieval methods include the wire-loop technique, balloon displacement, sling techniques, excimer laser sheath ablation, and rigid forceps dissection. Rigid forceps (endobronchial, bronchoscopic, or laparoscopic) introduced through large-bore sheaths (14-26 Fr) allow precise dissection of the neointimal cap covering the filter apex and provide strong countertraction to free embedded struts [3, 5]. Evidence reports technical success with rigid forceps even in severely tilted or long-dwelling filters (52.3 vs 18.5 months), with major complication rates of only 1-2% [5]. Laser sheath ablation achieves slightly higher success (98.1%) for the most chronic implants but requires specialized equipment and carries a similarly low risk of caval injury or component embolization [5].

Advanced retrieval techniques have low major complication rates, though they are higher than standard methods and include IVC dissection, pseudoaneurysm, strut fracture with embolization, and retroperitoneal hemorrhage [3]. Most events are manageable endovascularly with balloons, stents, prolonged balloon tamponade, or additional snaring [3, 6]. The availability of rigid laparoscopic or similar graspers in operating rooms makes forceps-based retrieval particularly practical when snare, ureteroscopic forceps, and balloon methods fail, while still avoiding open surgery [7].

This case of a 43-year-old male demonstrates the safety and efficacy of rigid laparoscopic forceps as a bail-out tool for complex embedded IVC filter retrieval, adding to the growing experience with rigid grasping devices in challenging cases.

## Case presentation

A 43-year-old male with a history of bronchial asthma, previous laparoscopic sleeve gastrectomy, and laparoscopic cholecystectomy was referred for elective retrieval of an inferior vena cava (IVC) filter. Notably, the patient had no documented venous thromboembolism at the time of filter placement. Anticoagulation was deferred due to the combination of recent cholecystectomy (risk of postoperative bleeding) and an incidentally discovered IVC injury sustained during cholecystectomy, which the operating surgeon judged to be at risk for expansion with systemic anticoagulation. The filter was therefore placed as a prophylactic measure. In retrospect, this indication was marginal, and we acknowledge that the filter may not have been strictly necessary according to current guidelines.

The filter had been placed four months earlier (119 days) via the right common femoral vein. Pre-retrieval imaging was not repeated, but the original placement venogram had shown a suprarenal position.

The patient was admitted electively for IVC filter removal under general anesthesia in a hybrid operating room. Right internal jugular vein (IJV) access was obtained under ultrasound guidance. A guidewire was advanced, and a catheter was positioned in the infrarenal IVC. Check angiogram revealed that the filter arms were located inside the right renal vein, and the filter hook was embedded into the IVC wall.

**Figure 1:**
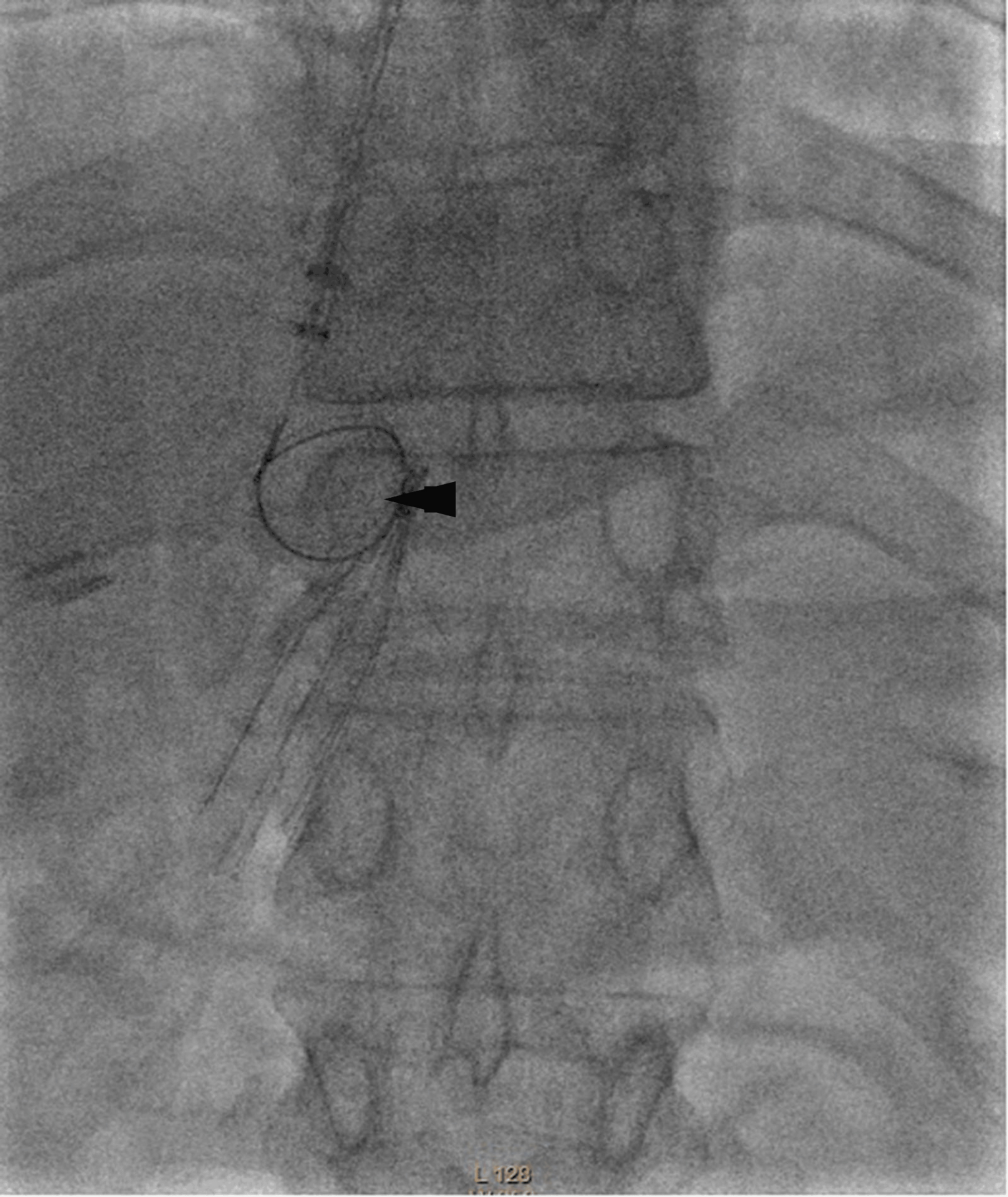
Failed snare retrieval attempt Snare (arrowhead) unable to engage the filter due to hook embedment in the IVC wall and arm protrusion into the right renal vein.

**Figure 2:**
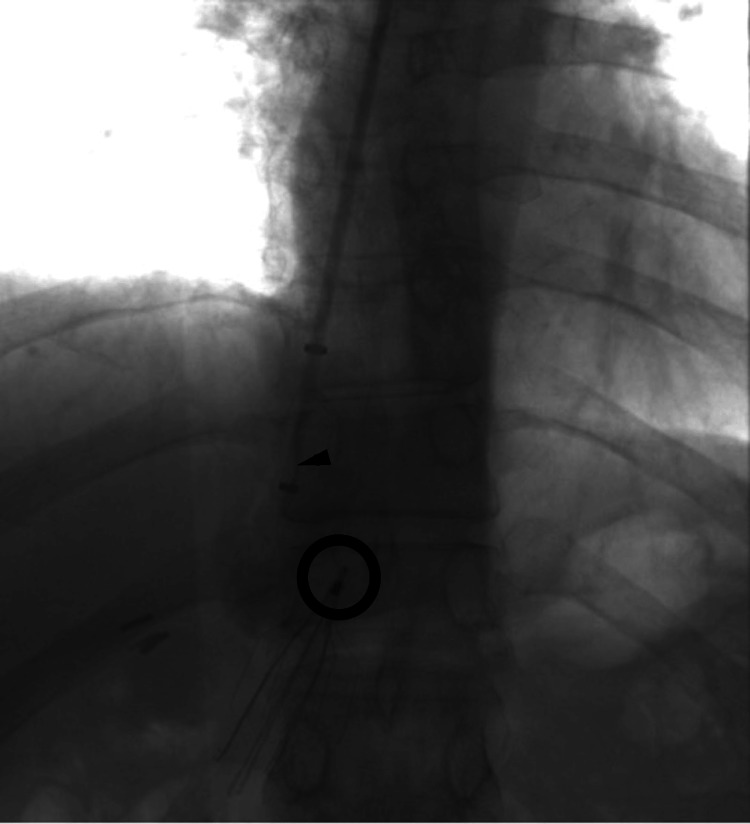
Ureteroscopic forceps attempt Flexible ureteroscopic forceps (arrowhead) unable to generate sufficient countertraction against the embedded filter hook (open circle).

Initial retrieval attempts using conventional endovascular techniques were unsuccessful. Multiple efforts with a snare device failed to engage or dislodge the incorporated filter (Figure 1). The sheath was then exchanged for a larger access (20 Fr, then 26 Fr). Subsequent attempts with ureteroscopic forceps also proved ineffective, as the flexible instrument could not generate sufficient countertraction against the firmly embedded filter (Figure 2). Dissection with a Fogarty balloon likewise failed to mobilize the device.

**Figure 3:**
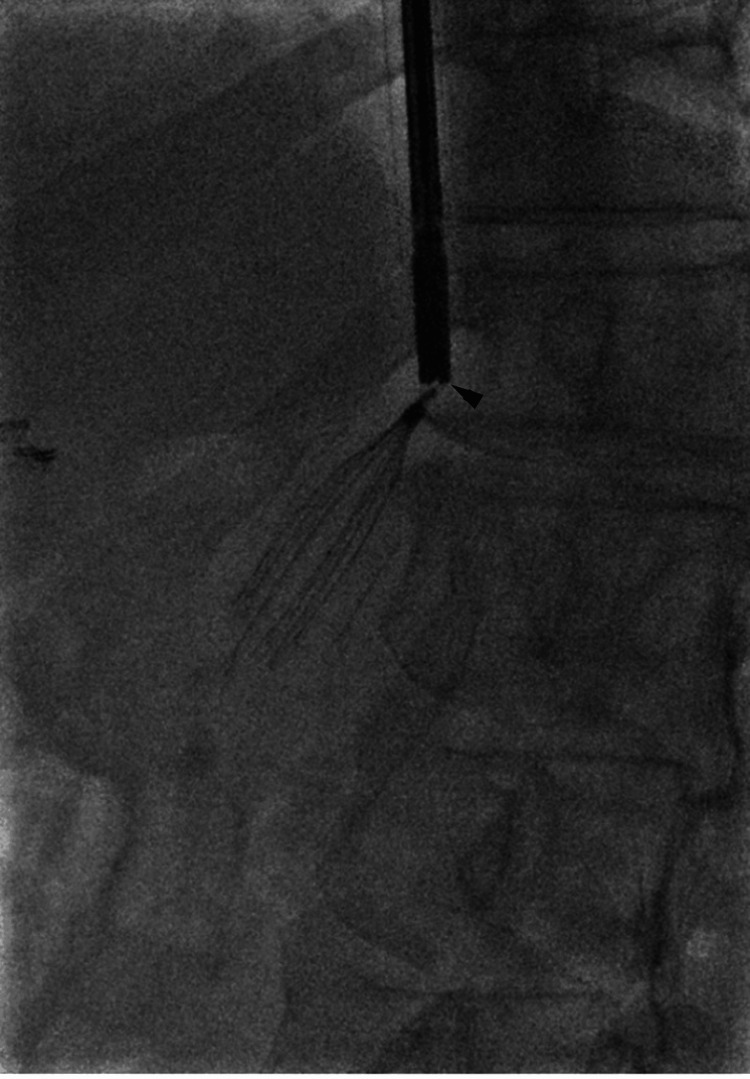
Rigid laparoscopic forceps engagement Rigid laparoscopic forceps (arrowhead) introduced through 20 Fr sheath securely capturing the filter apex.

**Figure 4:**
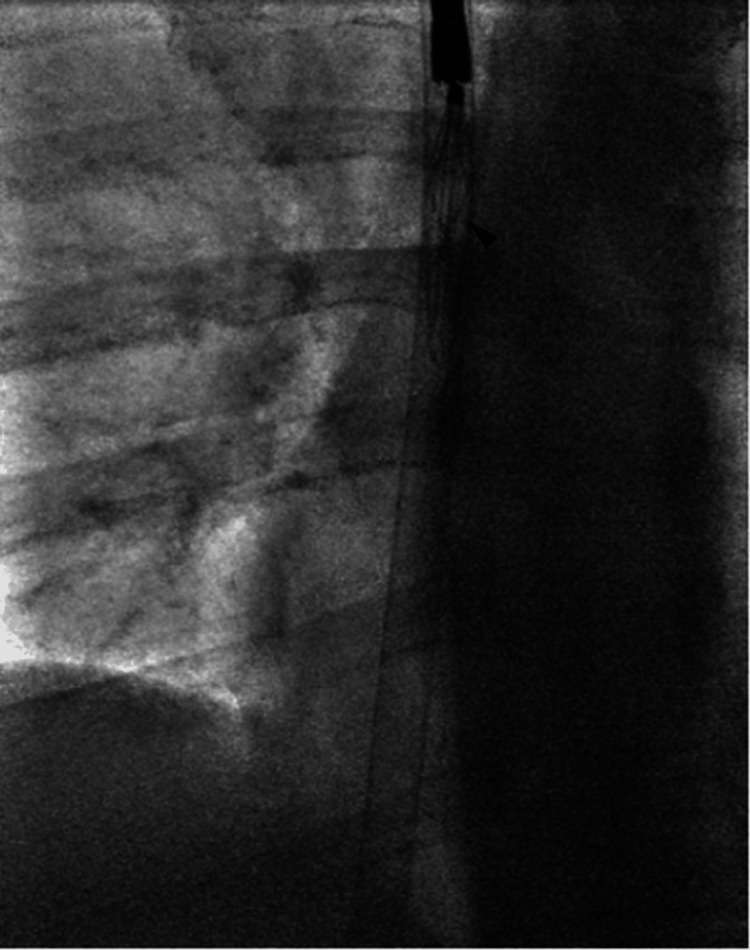
Filter removal Successfully retrieved IVC filter with intact struts (arrowhead) following sequential detachment from the caval wall.

Given the refractory embedding, the procedural strategy was modified to an advanced endovenous approach. Rigid laparoscopic forceps were introduced through the 20 Fr sheath. The robust jaws of the rigid grasper provided superior mechanical advantage, enabling secure capture of the filter apex and sequential detachment of the embedded hook and struts from the caval wall. The filter was successfully removed in toto through the sheath (Figures 3, 4).

Completion angiography demonstrated no extravasation. The puncture site was closed with 3-0 Monocryl suture, and a pressure dressing was applied. The patient tolerated the procedure well, remained hemodynamically stable throughout, and was discharged the next day in excellent condition without requiring analgesia.

This case highlights the technical difficulties of a deeply embedded IVC filter with arm protrusion into the renal vein and hook embedment into the caval wall. Rigid laparoscopic forceps served as a useful bail-out tool when standard and flexible endovenous retrieval techniques failed, thus avoiding the need for open surgical conversion.

## Discussion

This case demonstrates the successful use of rigid laparoscopic forceps for the retrieval of a deeply embedded IVC filter following the failure of standard snare techniques, flexible ureteroscopic forceps, and balloon dissection. Despite a relatively short dwell time of four months, strut endothelialization and hook incorporation into the caval wall occurred. We hypothesize that the prior iatrogenic IVC injury may have contributed to accelerated endothelialization, though direct evidence for this association is lacking. This highlights that embedding can occur quickly in specific clinical contexts, even before the "standard" high-risk period beyond six months [5].

Recent evidence indicates that prolonged dwell time is the strongest predictor of retrieval complexity due to progressive neointimal hyperplasia and strut penetration. However, our case shows that anatomical factors, such as filter arm protrusion into the renal vein, can similarly complicate removal despite shorter implantation times. In their systematic review of advanced IVC filter retrievals, Kethidi et al. reported a mean dwell time of 602.5 days across 770 attempts, with a pooled technical success rate of 92.6% and a major complication rate of only 2.8% [8]. In that analysis, rigid forceps techniques were 95.5% successful, compared with 98.8% for excimer laser sheath ablation, though the laser required specialized equipment not universally available [8]. Yu et al. demonstrated a laser success rate of 98.1% (636/648 patients) compared with 93.7% for forceps (342/365 patients), with major complication rates of 1.6% and 2.1%, respectively. Procedure and fluoroscopy times were slightly shorter with laser, but forceps remained highly effective and more widely available [5]. Our 100% success with rigid laparoscopic forceps mirrors these forceps outcomes but notably occurred with a much shorter dwell time.

The choice of rigid laparoscopic graspers in our case, rather than the more commonly reported endobronchial forceps, highlights a practical advantage in hybrid operating environments where laparoscopic instruments are readily available. The off-label use of rigid forceps (endobronchial, bronchoscopic, or laparoscopic) through large-bore sheaths (14-26 Fr) for dissection of the neointimal cap and countertraction on embedded struts has been validated, with success rates of 95% and major adverse events in less than 3% of patients [9]. Kuo et al. confirmed that forceps remain a cornerstone bail-out tool when snare or balloon methods fail, particularly for tilted or tip-embedded filters, with the added benefit of avoiding the photothermal tissue ablation required in laser techniques [10]. In contrast to laser sheath ablation, which excels with closed-cell designs and very chronic implants, forceps methods offer comparable efficacy at lower cost and without the need for capital investment in laser platforms [8].

It is important to acknowledge that rigid forceps retrieval carries a potential risk of irreparable vessel wall injury, especially in cases where filter struts are embedded into venous side branches such as the renal vein. The same principles that predispose to filter embedment - namely, neointimal hyperplasia and non-linear caval anatomy - also increase the risk of caval tear or perforation during forceful manipulation. Therefore, this technique should be reserved for cases where standard retrieval has failed, and operators must have a contingency plan, including balloon tamponade or surgical repair, in the event of caval injury.

We also acknowledge a fundamental limitation of this case. The original indication for filter placement - a minor, self-sealed IVC injury without documented venous thromboembolism or contraindication to anticoagulation - falls outside standard guideline recommendations. In retrospect, the filter may not have been necessary. This observation underscores the importance of multidisciplinary decision-making and adherence to established indications for IVC filter placement. Furthermore, the use of rigid forceps retrieval should ideally be performed in a hybrid operating room with immediate access to vascular surgery support, as bailout open repair would be required in the event of uncontrolled caval injury.

Similar cases further support the efficacy of rigid grasping devices in challenging embedded filters. A case report by Wang et al. described laparoscopic-assisted (not purely endovenous) retrieval of an 8-month embedded filter after failed interventional attempts, using direct laparoscopic dissection of thickened intima under visualization. The procedure was safe and avoided open conversion [11]. Liu et al. described two patients with Denali filters (dwell times 2 and 7 months) in whom standard and conventional forceps failed due to tip embedding and thrombosis. The authors employed a novel modification combining a wire-loop sling with simultaneous pushing via flexible endoscopy forceps on the filter shoulder, achieving successful retrieval without perforation or fracture [12]. Although our case used rigid laparoscopic jaws for direct apex capture rather than this push-pull modification, both approaches illustrate the adaptability of forceps-based strategies when initial attempts fail.

Complication profiles in contemporary cohorts remain favorable compared with historical data. Kethidi et al. found no procedure-related mortality and short-term major events (primarily caval dissection or strut embolization) in only 2.8% of advanced retrievals. These rates are higher than with standard techniques but are manageable endovascularly in most cases [8]. A systematic review by Rodriguez et al. noted that prolonged dwell time and complex techniques correlate with higher adverse events, yet overall rates remain below 5%, with most resolved by stenting or balloon tamponade [13]. Our uneventful recovery and intact completion angiogram are consistent with these low-risk profiles, even in a case with filter arm protrusion into the renal vein.

Despite technical advances, retrieval rates globally remain suboptimal. Even in dedicated follow-up programs, retrieval rates range from 40% to 74.5%, but real-world rates are substantially lower, with some cohorts reporting a median retrieval rate of only 6.2% within three months and 14.8% within one year [14]. Recent studies implementing automated EMR-integrated tracking programs have shown improved retrieval rates, emphasizing the importance of institutional protocols over technical expertise alone [15].

In the future, more widespread use of dedicated IVC filter clinics and emerging purpose-designed endovascular graspers may further reduce the need for off-label tools and open surgery. As retrieval programs mature and awareness improves, incorporation of such versatile rigid tools may further minimize the already rare need for open surgery and improve overall retrieval rates.

## Conclusions

This case demonstrates the safe and effective removal of a deeply embedded IVC filter using rigid laparoscopic forceps when standard snare, ureteroscopic forceps, and balloon techniques failed, despite a short dwell time of only four months. Although the procedure was technically challenging - with failure of snare, ureteroscopic forceps, and balloon techniques before successful retrieval - the patient experienced immediate discharge, full recovery, and no vascular injury. Rigid laparoscopic graspers are a feasible bail-out option in hybrid operating rooms, offering good mechanical advantages without requiring access to laser equipment or conversion to open surgery. This case suggests that rigid laparoscopic forceps may serve as a useful bailout technique in selected difficult cases, while acknowledging that broader conclusions require further experience and study. In the future, dedicated IVC filter follow-up clinics, improved tracking systems, and purpose-designed endovascular devices are likely to improve retrieval rates and further reduce the use of off-label devices while still ensuring excellent safety.
